# Open Innovation and Regulatory Challenges in New Modality Development: The Pivotal Role of Contract Development and Manufacturing Organisations in Advancing Antibody Drugs

**DOI:** 10.1007/s43441-024-00701-x

**Published:** 2024-10-28

**Authors:** Hiromu Yoshiura, Yayoi Kawata, Shintaro Sengoku

**Affiliations:** Department of Innovation Science, School of Environment and Society, Tokyo Institute of Technology, Tokyo, Japan

**Keywords:** CDMOs, MAbs, Partnership strategy, Innovation process, Technological platform

## Abstract

**Background:**

Ensuring regulatory-compliant manufacturing capability is an essential challenge for new treatment modalities, but its internalisation is not easy for pharmaceutical companies, especially start-ups. This study examines the functions and requirements of contracted development and manufacturing organisations (CDMOs) using the development process of antibody medicines as a case study.

**Methods:**

Utilizing PubMed, Cortellis and Patent Integration databases, this study delves into publication and contractual trends in monoclonal antibody drugs (mAbs) development, alongside an analysis of patent filings by CDMOs, offering a comprehensive overview of the evolving landscape in mAbs innovation.

**Results:**

In the early stages of mAbs development, dedicated bio firms (DBFs) led R&D with superior drug discovery technology but lacked manufacturing capability, which was complemented by CDMOs. This collaboration was an opportunity for CDMOs to expand their capabilities beyond manufacturing technology into antibody drug candidate discovery and structural optimisation technology. From mid-development onwards, it established a technology platform based on these capabilities and developed and established partnerships with existing pharmaceutical companies, including mega pharma.

**Conclusions:**

The impact of institutions and regulations on the innovation process was assessed during this development process. These findings are expected to provide valuable insights into the innovation system for new modalities.

## Introduction

The presence and importance of biopharmaceuticals have increased with the advancement of biotechnological technologies [1–3]. When conventional small-molecule compounds were the norm, closed innovation was the norm, with pharmaceutical companies working on R&D in-house and reinvesting profits from new drugs, including blockbusters, into new R&D [4, 5]. However, the productivity of R&D to create new drugs has been declining and has long since moved to external collaboration through licensing and acquisitions [6]. In particular, in the current environment of rapid development of new biotechnological modalities, such as antibody and nucleic acid drugs, open innovation beyond company boundaries is necessary to continue providing value to the market [7–9].

This study aims to understand the formation mechanisms of inter-organisational collaboration through open innovation in the acceptance and development of new modalities. Understanding the development process of a new modality enables the adoption of appropriate partnering strategies according to the situation, which can lead to a reduction in soaring R&D costs and an improvement in market valuation. Several giant pharmaceutical companies are actively adopting open innovation to reduce their R&D costs through appropriate open innovation [10]. In this study, the development process and mechanisms of the innovation system were investigated and analysed using the established modality, monoclonal antibody drugs (mAbs), as a case study. In particular, it focused on contracted development and manufacturing organisations (CDMOs) as key actors, explored their functional requirements and discussed the challenges and prospects in terms of regulatory science.

The mechanism of inter-organisational collaboration transition in the development of mAbs has been the subject of several studies, and Kong et al. investigated and analysed the development process of inter-organisational collaboration networks in the R&D of mAbs. The results showed that new organisations, including dedicated biotech firms (DBFs), played a central role in the early stages of the development of novel modalities such as mAbs [11]. The size and complexity of the collaborative network continuously increased as these DBFs established new partnerships with existing pharmaceutical companies. It has been reported that the establishment and evolution of open innovation in the mAbs industry led to a rapid increase in the number of mAbs entering clinical trials after around 1997 and continued to increase over the following decade [11, 12].

**Figure 1:**
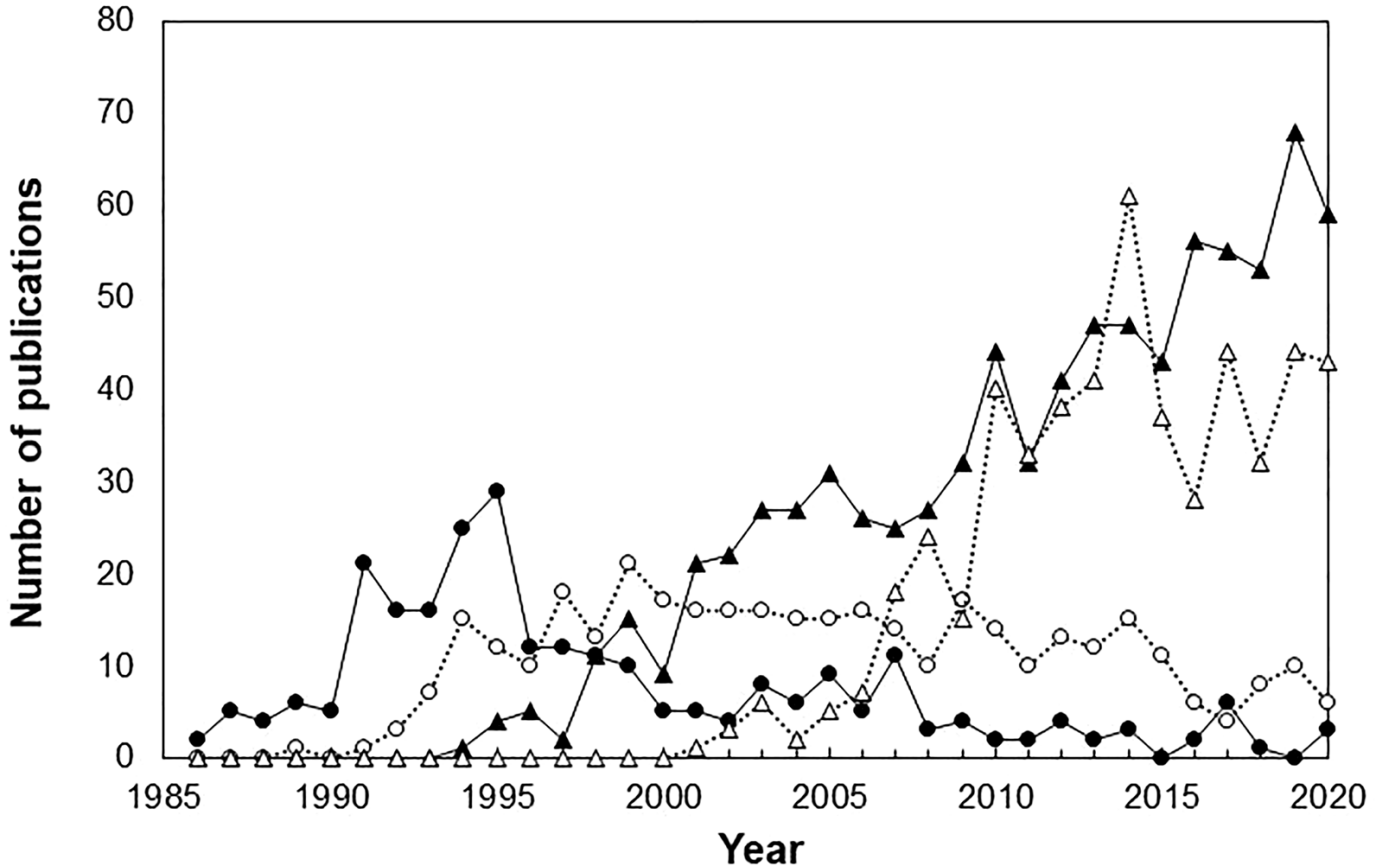
Publication Trends in the Clinical Development of mAbs. The Number of Scholarly Publications from 1985 to 2020 Related to the Clinical Development of mAbs were Illustrated. Each mAb Type, Murine (black Circle), Chimeric (white Circle), Humanized (black Triangle), and Fully Human (blank Triangle) were Plotted.

It has been reported that the rapid development of mAbs was contributed to by the emergence of fully human antibody production technology between the late 1980s and late 1990s [13]. Fully human antibody production technologies greatly improved immunogenicity, one of the most significant challenges faced by mAbs up to that time [14]. These technologies were developed by the DBFs and introduced into existing pharmaceutical companies through a process of open innovation. In practice, however, earlier antibody production technologies continue to operate (Fig. 1). It has been reported that in all mAbs used in clinical trials, technically previously established humanised antibodies are also treated as the dominant modality [15]. This indicates that triggers for the development of mAbs may exist beyond fully human antibody production technology.

Lack of manufacturing capacity was a major problem in the early development of mAbs, and CDMOs are likely to have contributed to solving this problem. Kurata et al. compared CDMOs in biopharmaceuticals with the foundry business in the semiconductor industry and reported that CDMOs, like foundries, are increasingly becoming reported an increasing presence as a technology platform for the production of mAbs [16]. Nishida et al. also targeted gene therapy and identified the importance of the manufacturing function in the novel modality [17]. Their findings reveal that although DBFs without manufacturing facilities can initiate clinical trials, at a certain stage they need to develop appropriate manufacturing and marketing strategies and invest in alliances and capital with external partners. It is assumed that this theory was also true for mAbs.

One point of observation is the development phase of the target mAbs product at the time the contract manufacturing agreement was signed. Another is the size of the company that contracted out the manufacturing. Observing these two points, we can infer what was required of the contractor. If a contract manufacturing agreement was signed in the early stages of development, the functions required of the contractor are likely to involve the development of the manufacturing process. On the other hand, if the contract is signed after the application has been submitted and approved, the contractor would be required to perform pure manufacturing functions, as it would not be easy to change the manufacturing process. In addition, the construction of mAbs plant requires a significant investment, generally above USD 100 million [18]. It is a significant risk, especially for small companies, to own their manufacturing facilities, and it is important to manage the risk by outsourcing manufacturing to CDMOs until the application and launch of the product. Therefore, research is underway on the process for making rational decisions to outsource manufacturing to CDMOs [19].

## Methods

PubMed search engine (http://www.pubmed.gov) was used to identify the number of publication trends in the clinical development of each type of mAbs. PubMed is the database perceived as both easy to use and efficient and used by many researchers in medical and life sciences commonly [20]. Using criteria of ‘murine’, ‘chimeric’, ‘humanized’ and ‘fully human’ with ‘monoclonal antibody’ in ‘title/abstract’, we searched the related publications. The publications retrieved from the search were analysed to eliminate duplicates, and the remaining ones were used to create a figure.

The Cortellis Competitive Intelligence database (Clarivate Plc), a private database, was used to identify the number and nature of contracts signed by CDMOs. Cortellis is a database dedicated to healthcare matters in the seven largest public authorities worldwide and contains 2.5 million articles and over 440 000 patent documents [21]. Keywords were entered in the ‘Technologies’ section of the database and limited to contracts for therapeutic antibody drugs. The information was narrowed down to those contracts whose ‘Agreement Type’ was ‘Drug-Manufacturing/Supply’ among those whose ‘Highest Status’ (at the time the contract was concluded) was Phase 1, Phase 2, Phase 3, Pre-Registration, Registered or Launched. After removing contracts involving non-profit organisations from the extracted contract information, changes in the number of contracts over time were checked. In order to identify contract information relating to pure antibody drugs, contract information relating to ADCs was excluded from the analysis.

Next, the business revenue information of the companies that commissioned the production, as stated in the extracted list of contracts, at the time the contract was concluded, was investigated using publicly available financial information and other sources. A total of 73 contracts were identified for which the business revenue information of the contracting company could be identified. The number of these contracts was categorised and graphed by the time the contract was concluded and the Highest Status (at the time the contract was concluded) of the drugs subject to the contract manufacturing agreement.

Information on contracts for antibody medicinal products concluded by the six leading CDMOs between 2001 and 2020 was then extracted. The extracted contracts were classified according to the ‘Agreement Type’ of the contract, and changes over time were checked. The agreement types classified were ‘Drug-Development Services’, ‘Drug-Development/Commercialisation License’, ‘Drug-Early Research/Development’, ‘Drug Manufacturing/Supply’, Patent-Non-Exclusive Rights and Technology-Other Proprietary.

Information on patent applications from the six major CDMOs was obtained from the private database Patent Integration (Patent Integration Co., Ltd.) Patent Integration is a tool for productive searches of technological trends and is a patent search and analysis service that provides access to a patent database containing over 45 million patent gazettes in Japan, the US, Europe and China, as well as advanced analysis support linked to the patent database from a web browser [Yamashita, 2010]. It is a patent search and analysis service that provides a patent database containing more than 45 million patent gazettes from Japan, the US, Europe and China, and advanced analysis support linked to the patent database from a web browser [22]. In the patent search function, a list of WO patents containing ‘antibody drugs’ in the ‘abstract + claim scope’ was extracted. After reading the claims of the extracted WO patents and excluding WO patents that are not related to therapeutic antibody drugs, each patent was classified according to its claim content into ‘Manufacturing of mAb technology’, ‘New mAb’, ‘Formulation technology’, ‘Optimisation of mAb structure technology’ and ‘mAb discovery technology’, and the number of WO patents filed by major CDMOs and the time-series changes in the types of patents are summarised in the graph. Note that patents filed by Boehringer Ingelheim GmbH were excluded from this study. Because it was difficult to distinguish the patents aiming for CDMO business and other activities as like their R&D activities since Boehringer is also one of the big pharmaceutical companies.

## Results and Discussion

### Quantitative and Qualitative Changes in Antibody Drug Commissioning Contracts

**Figure 2:**
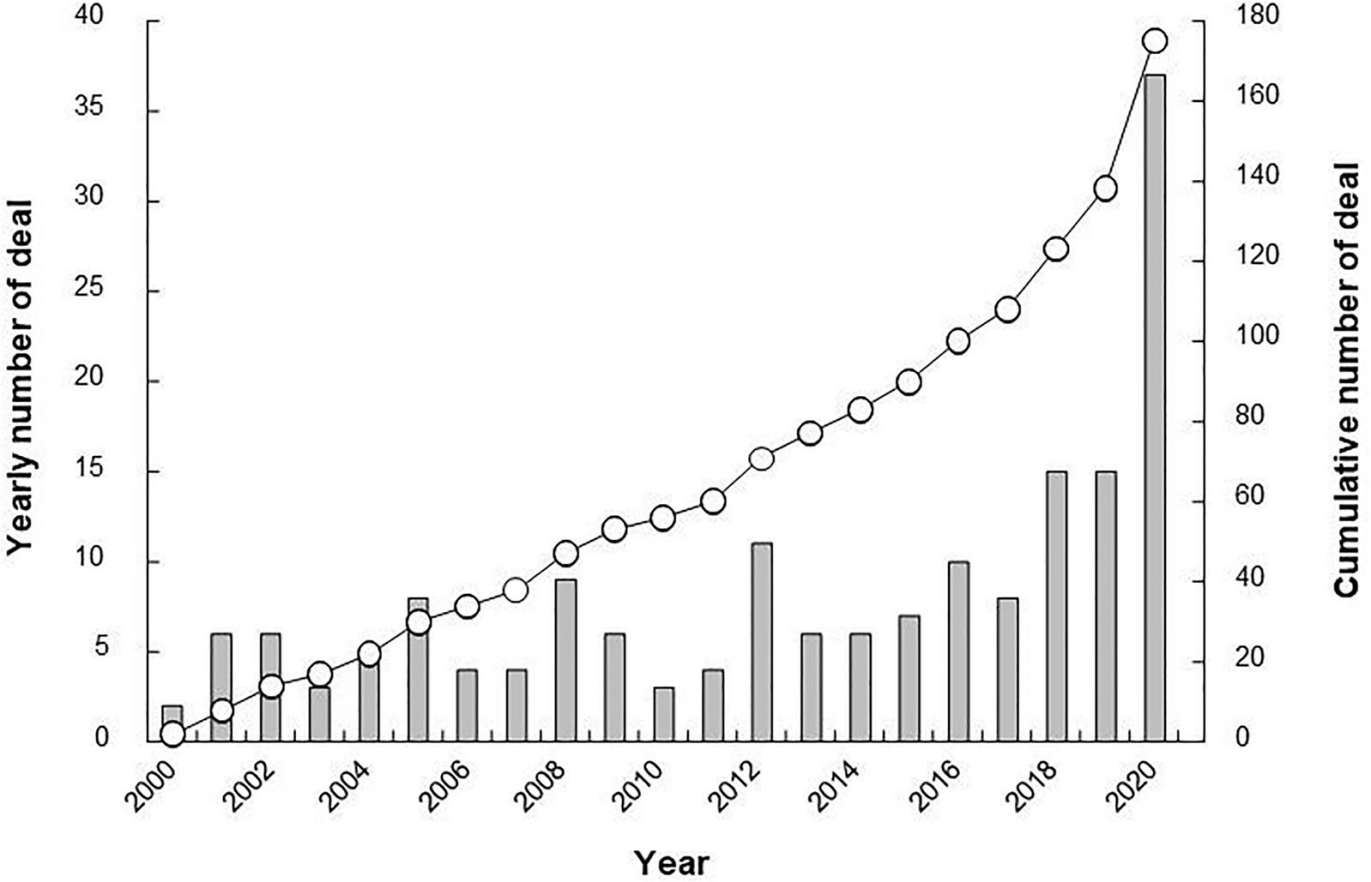
Trends in Contract Manufacturing Deals Related to mAbs. The Annual and Cumulative Number of Contract Manufacturing Deals from 2000 to 2020 Related to mAbs was Illustrated. The Bars and the Line Represent the Numbers of Annual and Cumulative Deals, Respectively.

An analysis using the Cortellis Competitive Intelligence database showed a yearly increase in contracts between companies for outsourcing mAbs production to CDMOs (Fig. 2). Especially in 2020, the number of contracts increased rapidly with the addition of the number of outsourced contracts for the rapid manufacturing of COVID-19 therapeutic mAbs. The results of the survey confirmed that 22 of mAbs subject to contract manufacturing agreements had reached the market as of 2020. This represents approximately 20% of the total number of mAbs launched in 2020, suggesting that CDMOs have played an important role in the development process of mAbs.

**Figure 3:**
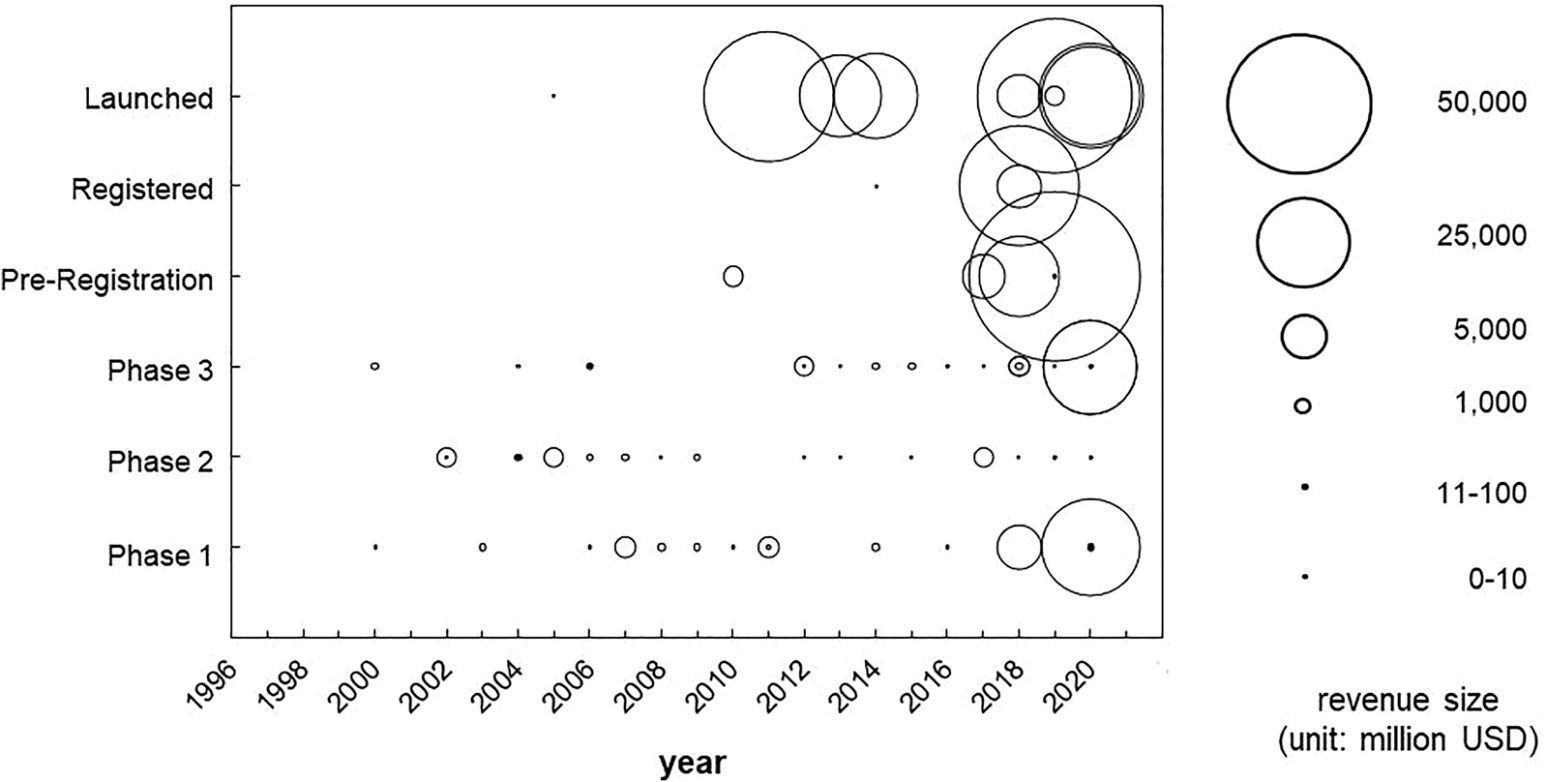
Distribution of Contract Manufacturing Deals Related to mAbs by Development Stage. Each Bubble Represents a Contract Manufacturing Deal with a Relative Size of Revenue of a Licensee. The Vertical and Horizontal Axes Indicate Development Stages of Drugs and Years of Transactions, Respectively.

Next, to identify the evolution of the roles/functions required of CDMOs, a survey and analysis of how the content of commissioning contracts has changed was conducted. Figure 3 presents the results of this analysis. First, the nature of contract manufacturing agreements changed after 2010. Prior to 2010, most requests were made by companies with revenues of USD 100 million or less and were predominantly Phase 1 or Phase 2 agreements. This is interpreted as the reason that in the early years of mAbs development, DBFs with annual revenues of less than USD 100 million were the main players, and the mAbs they created were the main focus. These DBFs had drug discovery technology, but owning manufacturing facilities was a risk, and it is thought that they outsourced their manufacturing, including the production of investigational medicines, to CDMOs.

Notably, since 2010, there has been a sharp increase in requests by companies with revenues of more than USD 5 billion. Existing pharmaceutical companies, including mega pharma companies, have ample funds and are therefore able to own their own manufacturing facilities. One of their objectives in outsourcing manufacturing to CDMOs would be to expand their manufacturing capacity. In addition, the fact that they often outsource manufacturing after the pre-registration phase makes it highly likely that they are outsourcing to accommodate adjustments in manufacturing volumes after launch.

Another important point is that, from 2010 onwards, some of the contracts for the clinical development phase can be identified as being outsourced by existing mega pharma companies. This is presumably because CDMOs have developed and internalised key technologies for the manufacture and development of antibody medicines, forming a comprehensive technology platform, which enables them to provide value to existing pharmaceutical companies beyond the expansion of their manufacturing capacity.

### Changes in the Capability of CDMOs

**Figure 4:**
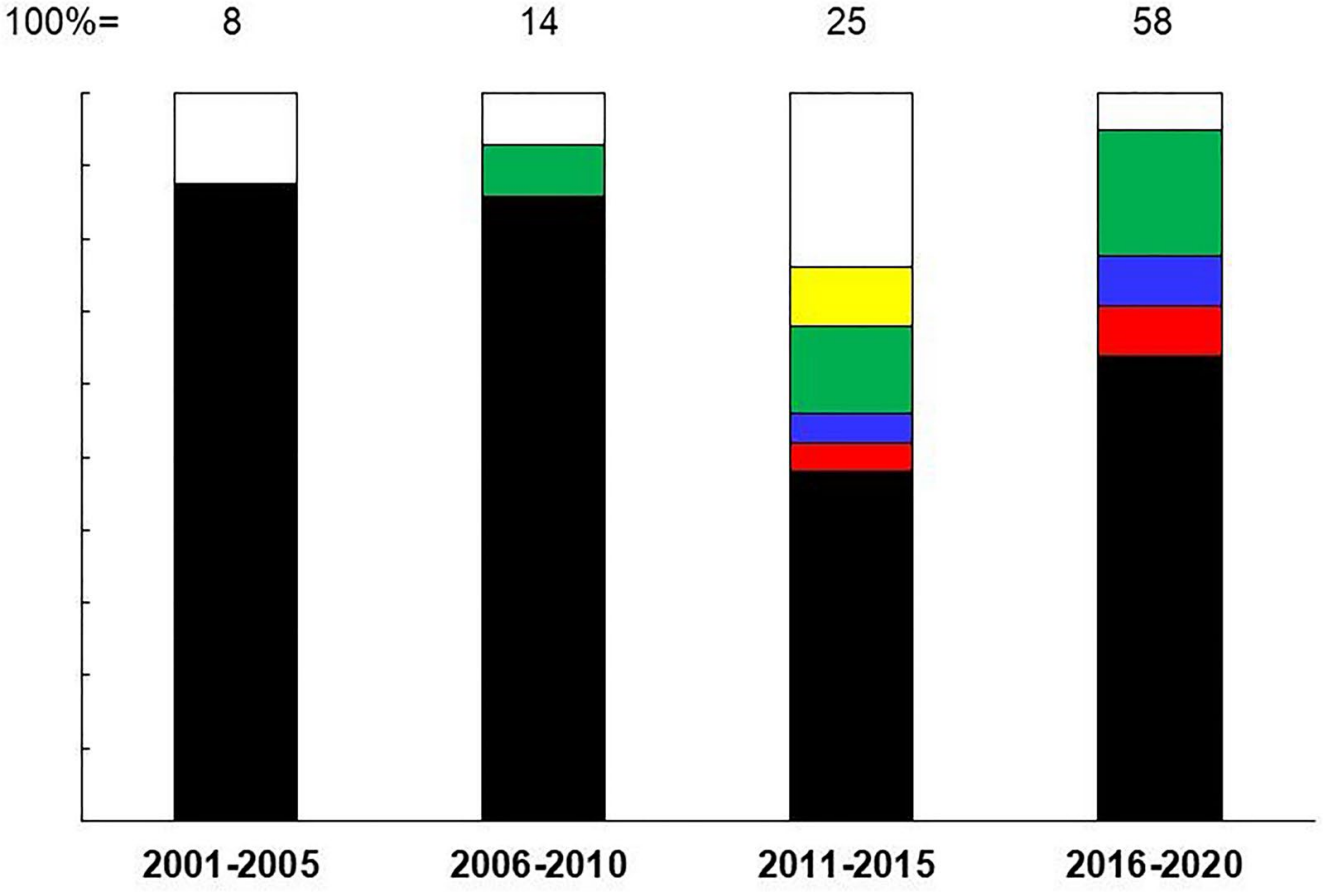
Changes in the Nature of Transactions Related to mAbs by Representative CDMOs. Types of Contracts Related to mAbs Undertaken by the Selected CDMOs (Lonza Group AG, Samsung BioLogics Co., Ltd., Boehringer Ingelheim GmbH., FUJIFILM Diosynth Biotechnologies, Wuxi AppTec, Inc. and AGC Biologics) were Illustrated, Divided into Four Periods as Shown. The Transaction Categories Encompass Manufacturing of mAbs (Black), Early Research/development (Red), Development/commercialization License (Blue), Development Services (Green), Non-exclusive Patent Rights (Yellow), and Other Proprietary Technologies (White).

In order to investigate why existing pharmaceutical companies are increasingly seeking to outsource to CDMOs, the capability of CDMOs and the changes they are undergoing were investigated and analysed. As representative CDMOs, Lonza Group AG, Samsung BioLogics Co, Ltd, Boehringer Ingelheim GmbH, FUJIFILM Diosynth Biotechnologies, Wuxi AppTec, Inc, AGC Biologics were investigated. The survey and analysis of the contract arrangements and their changes. Figure 4 revealed that until 2010, the contract arrangements were for contract manufacturing of antibody drugs. However, from 2011 onwards, contracts related to patent rights, technology-supply and development support have increased, accounting for about half of the total. This confirms the trend described in the previous section and indicates that the role of CDMOs in mAbs industry has changed since around 2010.

**Figure 5:**
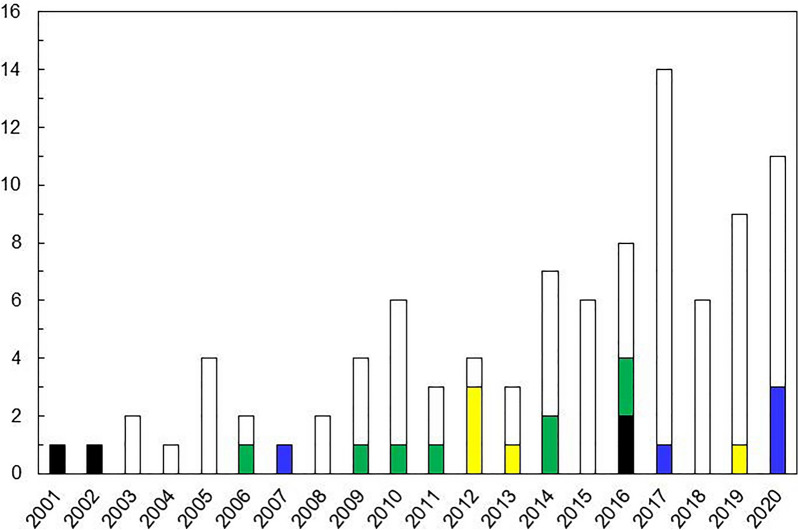
Trends in Patent Applications Related to mAbs by Representative CDMOs. Trends in PCT Patent Applications By the Selected CDMOs (Lonza Group AG, Samsung BioLogics Co., Ltd., Boehringer Ingelheim GmbH., FUJIFILM Diosynth Biotechnologies, Wuxi AppTec, Inc. and AGC Biologics) were Illustrated from 2002 to 2020. The Patent Categories Encompass Manufacturing of mAbs (White), New mAbs (Yellow), Formulation (Blue), Optimisation of the mAb Structure (Green), and the Discovery Of mAbs (Black). The Vertical and Horizontal Axes Represent the Number of Applications and Application Years, Respectively.

Figure 5 shows the number and type of international patents applied by the major CDMOs to mAbs. As shown in Fig. 3, before 2010, CDMO had mainly outsourced manufacturing from DBFs that possessed drug discovery technology but did not have their manufacturing facilities, so the capability in terms of manufacturing technology was sufficient. This shows the strategy of the CDMO industry as a whole, which was to develop the business by accumulating manufacturing experience, while at the same time gaining capability in upstream drug discovery research and development. The formation of a technology platform based on these efforts contributed to the expansion of partnerships with existing pharmaceutical companies in the early stages of development, and is considered to have earned the company an important position in the value chain for the development and manufacture of mAbs today.

### Triggers That Prompted Functional Changes in CDMOs

Based on these series of results, the present study identifies the role of CDMOs in the development process of mAbs and the evolution of their function in the industry bird’s eye view. Three triggers that prompted the functional change of CDMOs are considered to have been responsible for the change in the function of CDMOs.

The first trigger was the FDA Modernisation Act, which came into force in 1997 [23]. This Act consolidated biopharmaceutical product and manufacturing applications into one document, the BLA; before 1997, switching or expanding manufacturing facilities after an application required a new application. Furthermore, the FDA regarded CDMOs as part owners of the product, so for pharmaceutical companies to use CDMOs, sensitive data had to be shared and the name of the CDMO also had to appear on the product packaging, which greatly discouraged the willingness to use CDMOs [24].

The second trigger was M&A of DBFs by established pharmaceutical companies: between 2004 and 2009, a wave of acquisitions of DBFs with important antibody drug discovery technologies and developments by established pharmaceutical companies occurred [11]. During this period, many established pharmaceutical companies were facing patent expirations, pipeline phenomena, and cost pressures, and were looking for ways to improve their profit margins. They were also being squeezed by generic competition and needed strategies to improve research productivity and reduce costs. One of the strategies they took was the acquisition of DBFs for a comprehensive inclusion of their biopharmaceutical pipeline [25]. There are many reasons why pharmaceutical companies steered their acquisitions of DBFs. One of the most important reasons is that mAbs were more difficult for generic manufacturers to copy than chemically derived active pharmaceutical ingredients. Because their molecular mechanism of action depends on multiple domains, regulatory approval of mAbs biosimilars is subject to specific science-based guidelines and high development costs [26]. This may have accelerated the acquisition of DBFs by existing pharmaceutical companies. This made it important for CDMOs to take on contract manufacturing from existing pharmaceutical companies in addition to DBFs.

**Figure 6:**
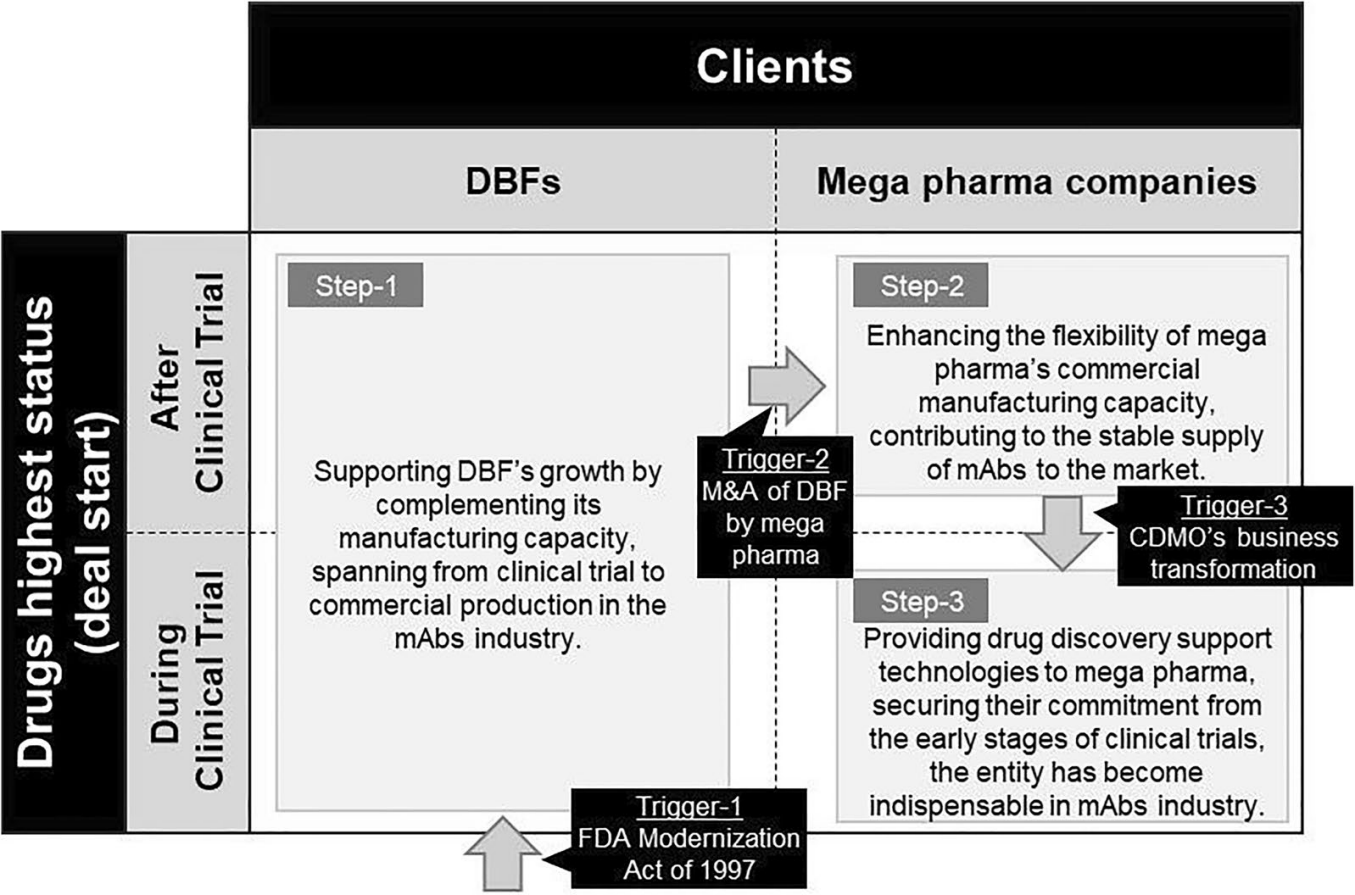
The Historical Evolution of CDMOs. Historical Stages (Steps 1 to 3) of CDMOs and triggers of their Transitions (Triggers 1 to 3) were Schematically Illustrated. The Services are Targeted at Clinical Development and Subsequent Processes, while the Beneficiaries of the Services are Organized into DBFs and Large Scale-Pharmaceutical Firms (Mega Pharma Companies).

The last trigger is a functional change by the CDMO itself. While the first and second triggers are external factors, the last trigger is an internal factor that the CDMOs themselves have pulled in order to increase their value in the antibody drug industry. The evolution of the CDMO’s function and role in the antibody drug industry as a result of each trigger is shown in Fig. 6: Step-1 was as a development partner as a complement to the manufacturing capacity of the DBFs, Step-2 as a pure manufacturing capacity complement to existing pharmaceutical companies and Step-3 as a complement to existing pharmaceutical companies. Step 3 is a development partner of an existing pharmaceutical company. The findings reinforce the mechanism of the mAbs development process, as previous studies have described the important role played by DBFs, but with limited reference to CDMOs.

## Conclusions

Research on the development process of mAbs has mainly focused on technology and innovation management during the R&D phase and lacked a manufacturing perspective. Our study and discussion here draw on inter-organisational collaboration in development and manufacturing, as well as research and analysis of publications and patent information, to describe the processes and mechanisms that led CDMOs to establish their present-day presence. In the early stages of the development of mAbs, CDMOs complemented the manufacturing functions that were lacking in DBFs. Subsequently, CDMOs expanded their partnerships with existing pharmaceutical companies and established their business models, while at the same time striving to acquire capabilities other than manufacturing. Today, based on its technology platform, it has successfully established partnerships from the research and development phase. The findings will help to understand the innovation process not only in mAbs, but also in diverse new modalities, and provide useful insights for various players in the pharmaceutical industry to develop their business strategies.

## Data Availability

PubMed search engine (http://www.pubmed.gov) was used to identify the number of publication trends in the clinical development of each type of mAbs. The Cortellis Competitive Intelligence database (https://access.cortellis.com/login?app = cortellis), a private database, was used to identify the number and nature of contracts signed by CDMOs. Information on patent applications from the six major CDMOs was obtained from the private database Patent Integration (https://patent-i.com/).
